# Medical student exam performance and perceptions of a COVID-19 pandemic-appropriate pre-clerkship medical physiology and pathophysiology curriculum

**DOI:** 10.1186/s12909-022-03907-5

**Published:** 2022-12-02

**Authors:** Melissa Chang, Andrew Cuyegkeng, Joseph A. Breuer, Arina Alexeeva, Abigail R. Archibald, Javier J. Lepe, Milton L. Greenberg

**Affiliations:** 1 School of Medicine, University of California, Irvine, USA; 2 Institute for Clinical and Translational Science, University of California, Irvine, USA; 3 Department of Neurology, University of California, Irvine, USA; 4 Department of Physiology and Biophysics, University of California, Irvine, Medical Sciences D350, CA 92697 Irvine, USA

**Keywords:** Remote learning, Virtual curriculum, COVID-19, Pre-clerkship, Undergraduate medical education, Physiology

## Abstract

**Background:**

Medical schools were compelled to abruptly transition pre-clerkship curricula to remote learning formats due to the emergence of the Coronavirus Disease 2019 (COVID-19) pandemic. We evaluated student perceptions of remote learning, exam performance, and utilization of third-party learning resources to assess the implementation of a newly developed pandemic-appropriate physiology curriculum.

**Methods:**

This was an observational study based on a survey conducted in the Spring of 2021 at the University of California, Irvine, School of Medicine (UCISOM). This study aimed to assess first (MS1) and second year (MS2) medical students’ perceptions of satisfaction, support, academic performance, and connectedness before and during the COVID-19 pandemic. The MS1 class began medical school during the first year of the COVID-19 pandemic, whereas the MS2 class did so prior to the start of the pandemic. A survey instrument was developed and validated to identify the impact remote learning had on student self-perceptions of the Medical Physiology and Pathophysiology course. Surveys were distributed to all students and responses were collected on a voluntary basis. Exam scores on a customized National Board of Medical Examiners (NBME) physiology shelf exam were also compared to objectively identify how the remote curriculum during the pandemic impacted academic performance.

**Results:**

Of 204 students enrolled, 74 responses were analyzed, with 42 MS1 (40% of MS1s) and 32 MS2 (31% of MS2s) responses. Overall, MS1s and MS2s were satisfied with the curriculum they received (95 and 97% respectively) and the school’s support of their concerns (86 and 100% respectively). Notably, only 50% of MS1s felt connected to their peers, compared to 94% of MS2s. Lecture attendance and self-perception of their academic performance were similar between both classes. Interestingly, the intra-pandemic class’s NBME exam average in 2020 (60.2% ± 8.9, *n* = 104) was significantly higher than the pre-pandemic class average in 2019 (56.8% ± 11.3, *n* = 100). Both classes primarily used course materials over third-party learning resources. An additional set of survey questions distributed only to the MS1 class found that the majority of MS1s reported minimal barriers with regards to accessibility, including internet connectivity, study-conducive environments, and balancing family commitments. Overall, pre-clerkship medical students had positive perceptions of the newly developed pandemic-appropriate physiology curriculum.

**Conclusions:**

Changes to the pre-clerkship physiology curriculum during the COVID-19 pandemic were met with overall satisfaction from the students and an increase in NBME scores. More attention to student connectedness is needed to improve how remote learning can be best optimized into future curricula development.

**Supplementary Information:**

The online version contains supplementary material available at 10.1186/s12909-022-03907-5.

## Background

Medical student education during the COVID-19 pandemic required rapid curricular innovations to maintain educational quality while adhering to distancing guidelines [1]. Medical schools transformed existing pre-clerkship curricular structures to remote synchronous and asynchronous learning, while maintaining critical in-person clinical education through the expansion of telehealth, personal protective equipment, and physical distancing [2]. While early evidence suggested that remote teaching was effective and preserved student learning and engagement [3], a recent review identified fewer clinical experiences and reduced case volumes as causes for concern [4].

While all medical schools were impacted by disruptions necessitated by institutional responses to COVID-19, medical students had varied experiences driven by the specific curricular changes adopted by each institution. For example, some medical schools maintained in-person anatomy laboratories [5–7], while others developed new, entirely-remote dissection courses for the first year of pre-clerkship training [8, 9]. Student feedback on pre-clerkship curricular changes also varied. For example, some institutions reported decreases in exam performance [7, 9, 10] and negative perceptions of course quality [9, 11], while others reported no change in exam performance [5, 6, 11] and positive perceptions of course quality [6, 12]. Here, we evaluate student perceptions and exam performance in the core pre-clerkship physiology and pathophysiology curriculum at the University of California, Irvine, School of Medicine (UCISOM).

In March 2020, UCISOM’s pre-clerkship Medical Immunology course was the first pre-clerkship course at UCISOM to be taught entirely through remote learning [6]. Course components, including lecture-based didactics, small group formative assessments, a self-directed learning module, and summative assessments, were successfully converted to online learning using synchronous videoconferencing with Zoom and asynchronous content delivery with Canvas and Panopto software. Student perceptions and performance following the abrupt transition to remote instruction were positive, with improved evaluations and no change in exam scores compared to the previous year [6]. Lessons learned from Spring 2020 served as a template to successfully adapt the first year (MS1) physiology curriculum into a remote learning format. The pre-clerkship Medical Physiology and Pathophysiology course was transformed into a new pandemic-appropriate structure over the summer of 2020 due to extensive institutional support and considerable input from rising second year medical students (MS2) [6]. Plans for the new course included remote components including synchronous didactics, expanded use of E-learning modules [13], and conversion of peer-led review sessions to online formats [14, 15]. Limited in-person, small-group sessions were maintained to preserve an electrocardiogram active learning activity [16, 17].

The goal of this study was to evaluate the implementation of the newly developed pandemic-appropriate pre-clerkship Medical Physiology and Pathophysiology course to aid in the generation of new hypotheses for continuous curricular improvement. To achieve this, exam performance was analyzed, and student feedback was obtained from a survey instrument distributed to MS1 and MS2 students.

## Methods

### Study population

This study included MS1 and MS2 students at the UCI SOM, a public institution that awards a Doctor of Medicine degree through a traditional four-year program enrolled during the 2020-2021 academic year. Medical students participate in the Medical Physiology and Pathophysiology course during the first semester of the MS1 year, which is integrated with core anatomy, histology, and doctoring courses. This study was qualified as exempt research by the UCI Institutional Review Board for Human Subjects, and student responses to survey questions were anonymous, shared only in aggregate form, with privacy and confidentiality maintained.

### Survey instrument design and implementation

To assess satisfaction, support, academic performance, and connectedness amongst students during the Medical Physiology and Pathophysiology course, we developed 30- and 22- question survey instruments for the MS1 and MS2 class, respectively. The survey was created using Qualtrics XM and distributed virtually via URL through class listserv, Canvas LMS, and a Slack page for UC Irvine medical students. Survey questions were categorized into different sections including “Satisfaction,” “Connectedness,” “School of Medicine Support,” “Academics,” “Resources,” and “Challenges with Remote Learning.” A 4-point Likert scale was used. A majority of survey questions were adapted from published surveys examining student perceptions of educational changes during the COVID-19 pandemic [12, 18–20]. “Connectedness” survey questions 5-8 were adapted from the Online Student Connectedness Survey (OSCS), a validated instrument to measure perceptions of connectedness of students enrolled in online programs in higher education [21]. We consulted faculty and students to establish face validity, using a similar process as described in prior studies involving medical student surveys [20]. For analysis of third-party educational resources in question 22 of the survey (Fig. 6), students were given answer choices based on resources widely used by pre-clerkship students at UCISOM per input from students from past years. Options included the textbook *First Aid for the United States Medical Licensing Examination (USMLE) Step 1*, Boards and Beyond (West Hartford, CT), Exam Master (Newark, DE), Zanki/Anki Flashcards, Osmosis (Evergreen, CO), Ninja Nerd (Bethlehem, PA), UWorld (Dallas, TX), and None. The instrument’s reliability was found to be high (Cronbach *α* = 0.90 for the MS1 survey; Cronbach *α* = 0.84 for the MS2 survey).

The respective surveys were sent to all respective MS1 and MS2 students enrolled at UCISOM in 2020-2021 (102 MS1s and 102 MS2s). The intra-pandemic MS1 class took the physiology course remotely, as the COVID-19 pandemic forced the curriculum to be shifted online. The pre-pandemic MS2 class surveyed in this study was the most recent class to have the traditional, in-person curriculum. The MS1 questionnaire had additional questions to reflect this fact and included topics regarding remote learning, such as contributing environmental factors that may have affected virtual learning. The survey remained open for the duration of March and April 2021, following completion of the Medical Physiology and Pathophysiology course and the first semester of the pre-clerkship curriculum. Only complete submissions of the survey were analyzed. A total of 74 complete survey responses were received and analyzed, with 42 responses from MS1s, representing 40% of the MS1 class, and 32 from MS2s, representing 31% of the MS2 class.

### Statistical analyses

Descriptive statistical analysis of counts/frequencies for each question and question category were calculated using Microsoft Excel Version 2020 (Microsoft Corporation, Redmond, WA, USA, Supplemental Table). Because the purpose of the study was to investigate student perceptions of two different types of preclinical curricula (pre-pandemic vs. intra-pandemic) rather than a hypothesis-driven study, formal power calculations were not used. Student responses were gathered over a 2-month period and included MS1 and MS2 students that had finished the pre-clerkship physiology curriculum at UCISOM. Exam scores were analyzed by the two-tailed, independent t test, using GraphPad Prism 9 (San Diego, CA); a *p* value of less than 0.05 was considered statistically significant. Survey instrument reliability was evaluated by calculating Cronbachs’s alpha (*α*) for both MS1 and MS2 surveys.

### Ethical considerations

To encourage student participation and honest feedback, student anonymity was protected by several methods. Survey responses were not linked to student names or other identifiable information, and there was no option to track which students had or had not completed the survey, Additionally, students were informed that survey responses were anonymous, optional, and would not affect the grades/evaluations they received in the class.

## Results

A total of 74 students completed the questionnaire, with 42 responses from MS1s, representing 40% of the MS1 class, and 32 responses from MS2s, representing 31% of the MS2 class.

### Comparison of student perceptions of curricular satisfaction and interconnectedness

**Fig. 1:**
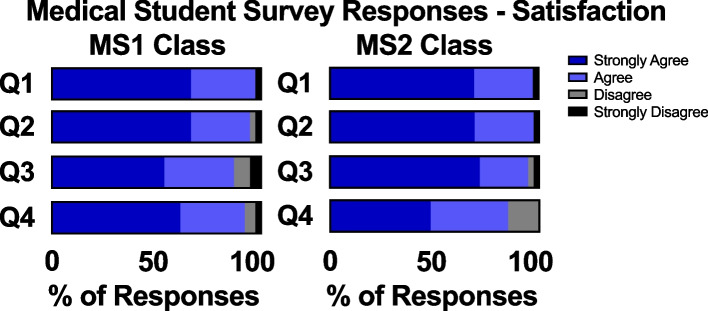
MS1 and MS2 students’ survey responses on student satisfaction. I am satisfied with the: Q1) quality of the pre-clerkship education that I have been receiving; Q2) physiology education I received in the PHYSIO 543AB: Medical Physiology and Pathophysiology course. The PHYSIO 543AB: Medical Physiology and Pathophysiology course: Q3) prepared me for the USMLE Step 1 exam in a way that was appropriate for the first semester of the MS1 curriculum; Q4) prepared me for clinical rotations in a way that was appropriate for the first semester of the MS1 curriculum

Overall satisfaction with the quality of the pre-clerkship education was comparable between the pre-pandemic MS2 class and the intra-pandemic MS1 class. 97% versus 95%, respectively, reported that they agreed or strongly agreed with the first survey question regarding satisfaction with the quality of the curriculum. However, a larger percentage of the MS2 class expressed strong satisfaction (41%) with the pre-clerkship curriculum as compared to the MS1 class (31%). 100% of students in both the pre- and intra-pandemic classes reported either being satisfied or strongly satisfied with the quality of the pre-clerkship Medical Physiology and Pathophysiology course. In addition, a similar proportion for students in both classes indicated that they either agreed or strongly agreed that the medical physiology course prepared them well for both USMLE Step 1 and third-year clinical rotations (Fig. 1).

**Fig. 2:**
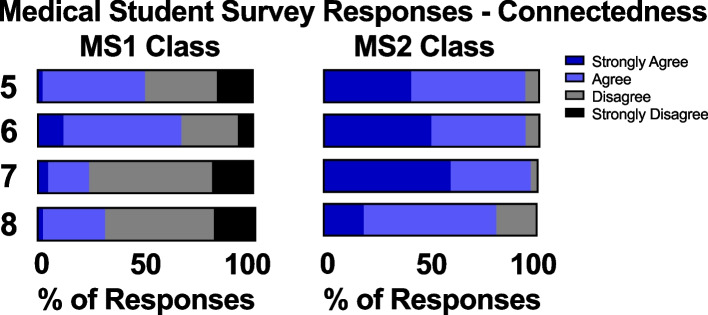
MS1 and MS2 students’ survey responses on connectedness. I felt: Q5) connected to my peers; Q6) that I had a good support system with my peers; Q7) that there were adequate opportunities for me to meet my peers; Q8) connected to faculty

At UCISOM, student perceptions of connectedness emerged as the most dramatic differences between the intra-pandemic MS1 class and the pre-pandemic MS2 class. Fewer students in the MS1 class agreed or strongly agreed that they felt connected with other members of their class as compared to the MS2 class (50% versus 94%, respectively). Following a similar trend, only 67% of the intra-pandemic MS1 class felt that they had a good support system with their peers, as compared to 94% of the pre-pandemic MS2 class. Unsurprisingly, the MS1s were less likely to agree or strongly agree that there were adequate opportunities to socially interact with other numbers of their class, with only 24% of the MS1 class agreeing or strongly agreeing that there were adequate opportunities as compared to 97% in the MS2 class. Likewise, only 31% of the MS1 class felt that they were connected with the faculty during the pandemic era as compared to 81% of the MS2 class during the pre-pandemic period (Fig. 2).

### Comparison of student perceptions of institutional support and academic performance

**Fig. 3:**
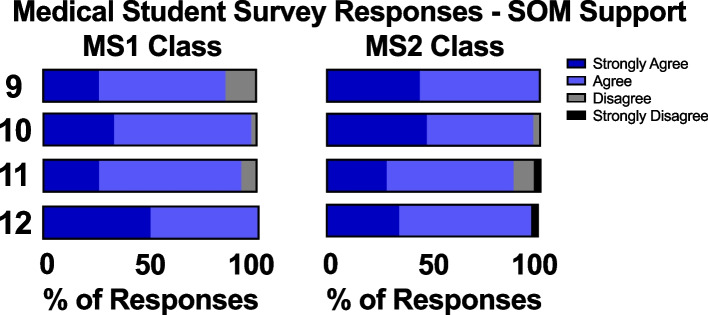
MS1 and MS2 students’ survey responses on School of Medicine support. I felt that: Q9) there were systems set in place for our class to express their thoughts and concerns; Q10) our class’s opinions were listened to. Q11) I feel supported by my school. Q12) I am aware of the mental health and wellness resources provided by our school

Regarding student perceptions of institutional support from the School of Medicine, a large proportion of both MS1s and MS2s agreed or strongly agreed that there were adequate systems set up for students to express their thoughts and concerns (86% for the MS1 class versus 100% of the MS2 class). A similar proportion of students in each class felt that their opinions were listened to by the school administration, with 98% of the MS1 class and 97% of the MS2 class indicating that they either agreed or strongly agreed. Similarly, there was little difference between the MS1 and MS2 classes regarding perceptions of support from the school and awareness of mental health and wellness resources provided by the school. Importantly, 100% of the intra-pandemic class agreed or strongly agreed that they were aware of the available mental health resources (Fig. 3).

**Fig. 4:**
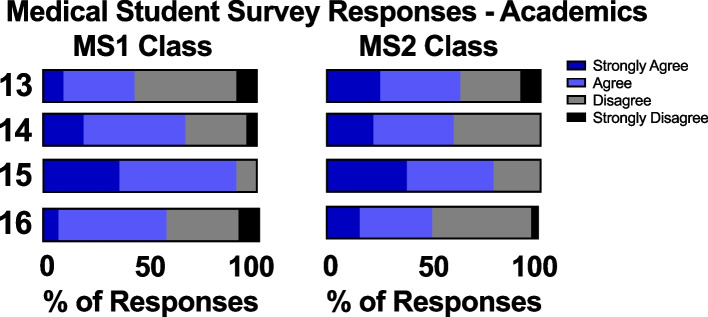
MS1 and MS2 students’ survey responses on academics. Q13) I find it difficult to concentrate on my studies. Q14) I feel like I have been performing optimally in my classes. Q15) I felt that watching lecturers (either live or recordings) was necessary for passing block exams. Q16) I tried my hardest on the physiology NBME

To evaluate student perceptions of their own academic performance, we compared survey responses between the pre-pandemic MS2 and intra-pandemic MS1 classes. 63% of MS2s agreed or strongly agreed that they had difficulty concentrating on their studies as compared to only 43% in the MS1 class. Overall, an equal portion of students in each class agreed or strongly agreed that they were performing optimally in their classes (66% for the MS1 class versus 60% for the MS2 class), regardless of in-person or virtual instruction. 91% of MS1s agreed or strongly agreed that course-provided lectures were necessary for passing class exams, with slightly fewer students (79%) agreeing or strongly agreeing with this in the pre-pandemic class. Lastly, virtual versus in-person education did not affect the amount of effort the students contributed to the end of course National Board of Medical Examiners (NBME) exam, with slightly over 50% of students agreeing or strongly agreeing that they tried their hardest in both the MS1 and MS2 classes (Fig. 4).

### Utilization of third-party learning resources and communication software

**Fig. 5:**
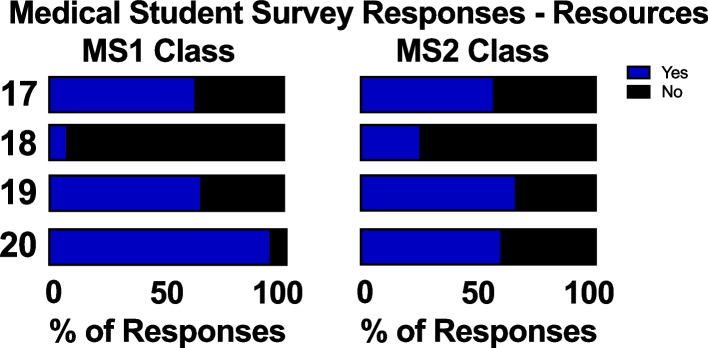
MS1 and MS2 students’ survey responses on study resources. I utilized: Q17) Third-party resources for learning; Q18) Third-party resources as my primary source of information; Q19) Third-party resources for practice questions; Q20) Software for communication regarding course content (i.e. Slack, Facebook, GroupMe)

**Fig. 6:**
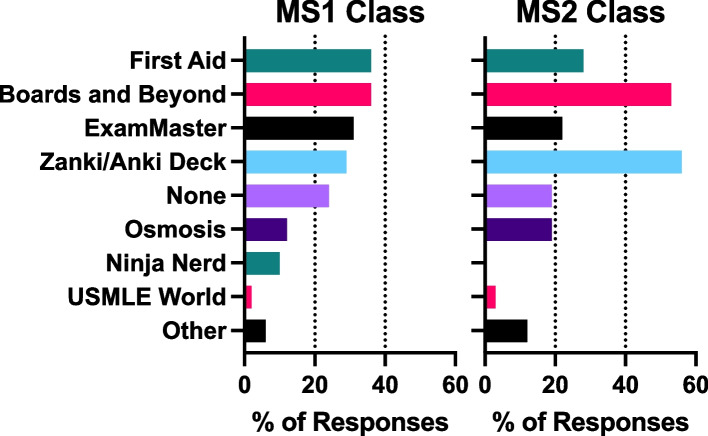
MS1 and MS2 students’ survey responses to Q21 regarding third-party learning study resources that they used, with sources utilized placed in ranking order. 6 students did not use any third-party sources for studying

While slightly more students in the MS1 class reported utilization of third-party learning resources compared to the MS2 class (62% versus 56%, respectively), fewer MS1s reported using third-party resources as their primary source of information compared to the MS2 class (7% versus 25%, respectively). Similar numbers of MS1s and MS2s report using third-party resources as a source of practice questions (Fig. 5). The classes differed in which resources they favored. The top three resources used by the MS1 class were First Aid (used by 36% of MS1 students), Board and Beyond (used by 36% of MS1 students), and ExamMaster (used by 31% of MS1 students). 29% of MS1s used Zanki (or another public Anki virtual flash card deck) decks for physiology review. Zanki/Anki decks, Boards and Beyond, and First Aid were the top three sources used by the MS2 class in 2019: 58% of MS2 students used Zanki/Anki, 53% used Boards and Beyond, and 28% used First Aid to study for the physiology course (Fig. 6).

Students the intra-pandemic curriculum utilized software to communicate with their classmates to a greater degree than students in the pre-pandemic curriculum. 93% of the MS1 class used software for communication regarding course content, compared to 59% in the MS2 class (Fig. 5). Preference for virtual communication software also differed among the MS1 and MS2 class. MS1 students primarily communicated via Slack (100%), Facebook (21%), email (2%) and texting (2%). MS2 students only reported using GroupMe (59%) and Facebook (50%).

### Comparison of synchronous attendance and NBME exam performance

**Fig. 7:**
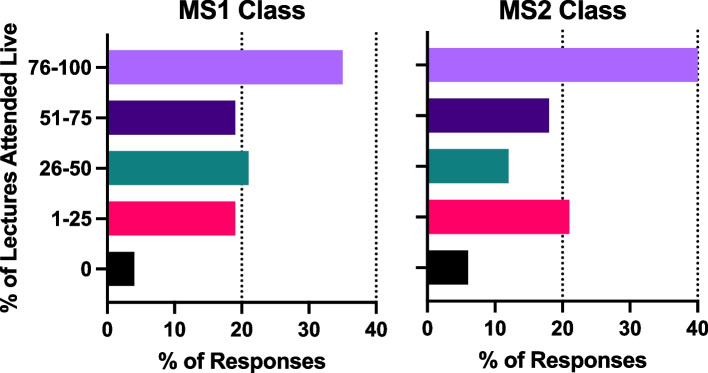
MS1 and MS2 students’ survey responses to Q22: Self-reported percent of lectures attended live. MS1 students attended didactic lectures in a synchronous format using Zoom software, and MS2 students attended live in the classroom, before the COVID-19 pandemic

Self-reported attendance of non-mandatory live, synchronous physiology lectures was comparable between the MS1 and MS2 class. 36% of MS1 students and 41% of MS2 students reported attending the majority of physiology lectures (76-100% of lectures), while 19% of MS1 students and 22% of MS2 students reported only attending a few physiology lectures (1-25% of lectures). In both groups, 5-6% of students reported attending no live physiology lectures (Fig. 7).

**Fig. 8:**
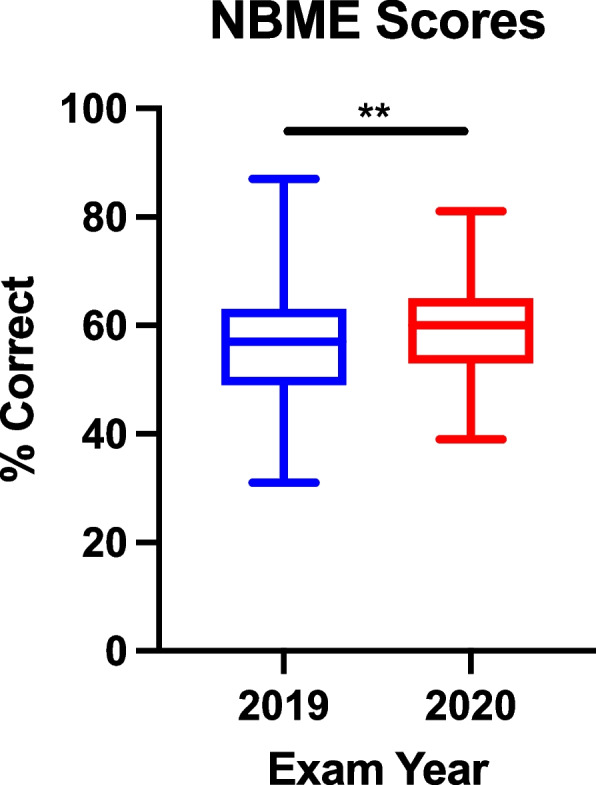
Customized Physiology NBME scores of pre-pandemic students in 2019 and intra-pandemic students in 2020. ** = *p*-value = 0.019. Whiskers represent min and max. *n* = 100 for MS2 class, *n* = 104 for MS1 class

The Medical Physiology and Pathophysiology course utilizes a customized Physiology NBME examination as a final exam that counts for 15% of the overall grade. The custom exam contains 75 questions pooled from the NBME question bank that are tailored to the topics emphasized in the course. The exam composition was identical for both the pre-pandemic MS2 class and the intra-pandemic MS1 class. The MS1 class scored significantly higher than the MS2 class on the Physiology NBME (*p* = 0.019, independent t test). The MS1 class average was 60.2% ± 8.9 (*n* = 104) in 2020, while the MS2 Physiology NBME class average was 56.8% ± 11.3 (*n* = 100) in 2019 (Fig. 8).

### Challenges with remote learning

The majority of MS1 students reported that they encountered minimal barriers with regards to accessibility to the online physiology course material. 98% of students agreed or strongly agreed that the devices (iPad, computer, tablet, etc.) they used for remote education were adequate for learning the material. 79% of students agreed or strongly agreed that living with family/roommates did not overly distract from their remote education. 69% of students agreed or strongly agreed that the online format of remote education did not detract from their education. 90% of students reported that they had a quiet, comfortable place to attend class and to study, while 10% of MS1 students reported having financial difficulties obtaining a reliable Internet connection at home.

The MS1 class overall had a positive perception of varied teaching styles that were utilized during remote learning, despite concerns about its interactivity. Only 55% of MS1 students reported feeling the remote learning experience was interactive, though 91% reported being able to ask questions during remote lectures if needed. 98% of students reported that online instruction courses were flexible with their schedules.

## Discussion

Our findings indicate that the new pandemic-appropriate physiology curriculum developed in the Summer of 2020 as a collaboration between students, faculty, and administrators [6] was successfully implemented in the Fall of 2020 with high levels of student satisfaction (Fig. 1) and improved academic performance (Figs. 4 and 8). Satisfaction with pre-clinical education was comparable between the two classes, indicating that the online nature of the curriculum didn’t significantly detract from students’ preclinical experiences. The results also show that the quality of teaching did not suffer, as both classes reported being similarly prepared for the USMLE Step 1 exam. This may be due to the supplemental training UCI faculty underwent for online teaching and navigating Zoom. Course coordinators were also present at every online lecture to help troubleshoot technological errors and record lectures that were promptly uploaded for asynchronous viewing.

**Fig. 9:**
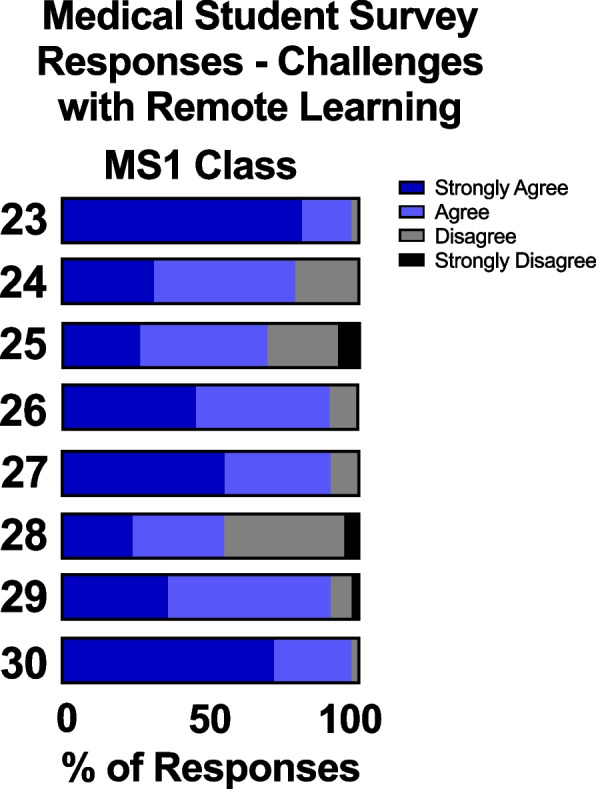
MS1 students’ survey responses on challenges with remote learning. Q23) The devices (iPad, computer, tablet, etc.) I used for remote education were adequate for learning the material. Q24) Living with family/roommates did not overly distract me from my remote education. Q25) Adjusting to the online format of remote education did not detract from my education. Q26) I had a quiet, comfortable place to attend class and to study. Q27) I was able to obtain a reliable internet connection in my home without financial difficulty. Q28) I felt that the remote learning experience was interactive. Q29) I was able to ask questions during my remote lectures if needed. Q30) Online instruction courses were flexible with my schedule

MS1s found that remote online instruction was flexible with their schedules (Fig. 9), a finding that was shared with other medical students at other institutions [19, 20, 22]. Emerging evidence suggests that medical students may prefer remote learning despite the opposite sentiment from professors [23]. Asynchronous viewing of lectures at students’ own pace may help combat “Zoom fatigue” which is experienced when looking at the computer screen for an extended period [20]. Contrary to findings at other institutions [19, 24], almost all of the MS1 class reported only minimal barriers to transitioning to online learning, noting that they had a stable internet connection, a quiet place to attend class and study, and were able to strike a balance between household responsibilities and attending class (Fig. 9). However, half of MS1 students felt that online learning sessions could have been more interactive, which has been shown to be an important aspect for medical students’ learning [25].

Both the MS1 and MS2 class agreed that there were adequate systems set up for students to express their thoughts and concerns to the school and course administrators (Fig. 3). Due to the uncertain nature of the pandemic and the potential stressors that could arise, UCISOM faculty provided numerous mental health and wellness resources that could be accessed virtually. Students were sent monthly email reminders of their eight free sessions with the campus psychiatrist and a myriad of other counseling opportunities that were provided at no cost. It is possible that students were able to better utilize resources emailed to them by the university, as studies show an increase in response rate to university emails during the pandemic [26].

One of the biggest differences we found was that the intra-pandemic MS1 class felt less connected to both peers and faculty compared to the pre-pandemic MS1 class (Fig. 2). This result supports previous findings that mode of learning (online vs. in-person) affects social outcomes, such as engagement with peers and facilitators and establishing friendships [27]. Although the UCISOM offered several opportunities to connect with peers and faculty, such as orientation week activities of beach hikes, pet walks, peer-led yoga sessions, and creating small bubbles of students for the in-person doctoring and anatomy courses, students yearned for more. The pre-pandemic class self-reported high levels of interconnectedness, providing hope that further curricular modifications will foster a return to the level of connectedness reported pre-pandemic as pandemic restrictions begin to ease.

We found that while the use of software for communication regarding course content increased during the pandemic, utilization of third-party learning resources did not (Figs. 5, 6). UCISOM students used similar physiology-related study materials reported by other pre-clerkship programs [28]. More MS2 students reported using third-party resources as their primary tool for learning compared to the MS1 class (Fig. 5). This finding may be due to USMLE Step 1 changing from a three-digit scoring system to a pass-fail system. The intra-pandemic MS1s may have felt less inclined to start USMLE Step 1 preparation early on and may have prioritized other aspects of their medical education such as extracurricular activities [29]. With the previous three-digit scoring system, medical students may have chosen to forgo studying traditional lectures to instead focus on USMLE Step 1 material via third party resources [30].

To our knowledge, we report the first pandemic-appropriate, pre-clerkship curriculum that resulted in improved student performance on an NBME exam (Fig. 8). At UCISOM, the intra-pandemic MS1 class had a significant improvement in NBME physiology grades compared to the pre-pandemic class. This in contrast with a previous study demonstrating that social outcomes (such as engaging with peers and facilitators, contributing to the group, and making friends) correlate with learning outcomes [27]. Other pre-clerkship programs have reported decreases [7, 9, 10] or no change in exam performance [5, 6, 11] during the pandemic, suggesting that widespread academic dishonesty is unlikely to have driven the increase in scores. UCI students also did not report a difference in the amount of effort they put into the end of course NBME exam, with slightly over 50% of students agreeing that they tried their hardest in both the intra-pandemic MS1 and pre-pandemic MS1 class (Fig. 4). The increase in NBME physiology grades seen in the UCI intra-pandemic course can be explained by several factors. First, the anatomy class that was held concurrently with the physiology course was changed from dissection-based to pro-sections performed by anatomy staff, decreasing the amount of time that students spent in the anatomy lab. This change was made to minimize in-person time during the pandemic, but it also increased the amount of time students had to dedicate to other studies, such as the Physiology course. Second, the expanded use of peer-led tutorials during the pandemic improved student understanding of physiological concepts [15]. Third, the intra-pandemic class was the first class to have the USMLE Step 1 exam as pass/fail instead of a numeric score, which may have impacted how they approached class exams and board exams. Lastly, while the majority of intra-pandemic and pre-pandemic classes reported usage of third-party study resources, the classes differed in which resources they favored (Figs. 5, 6). The increase in NBME scores supports that some pandemic-necessitated curricular innovations were beneficial for students’ academic performance.

### Suggestions for future years

Students agree that UCISOM has been attentive to student concerns and has quickly implemented appropriate changes to address those concerns (Fig. 3). Because the ongoing pandemic continues to create a sense of uncertainty among medical students, it is imperative that the school continues to solicit student feedback and respond appropriately. Greater efforts should be made to build a stronger sense of community in current and future medical student cohorts as the pandemic has significantly impeded the connectedness felt between students (Fig. 2). As pre-clerkship courses gradually transition back to in-person formats, considerations should be made to incorporate virtual elements into the curriculum, forming a hybrid format that bridges the advantages of in-person and remote education.

## Limitations

This study’s single-center analysis limits the generalizability to other institutions. In addition, because this study focuses on medical students, the results may not apply to students in other health professions. Since the survey was entirely optional and less than half of the pre-clerkship students responded to the request, survey responses many not be representative of the entire student body. There may be a response bias in which only those who felt most strongly about the curriculum provided a response. The MS2s were asked to reflect on their experiences from the previous year, which may have caused recall bias compared to the MS1s who reflected on a more recent experience. Finally, the response to the pandemic has been heterogeneous between medical schools due to factors such as government and institutional guidelines, temporal differences in peak COVID-19 case numbers, and technological integration which may not reflect current perceptions of medical students.

## Conclusions

The pandemic-appropriate pre-clerkship physiology curriculum achieved similar levels of success in student satisfaction and support compared to the pre-pandemic curriculum. Perceived academic performance was also comparable between the 2 years, and scores increased on the physiology NBME exam following implementation of the pandemic-appropriate curriculum. However, remote learning had a negative impact on student connectedness as students felt isolated and disconnected from their peers. Given the social aspects of in-person curricula and the increasing dependence on technology, a hybrid-model for medical curricula is urgently warranted to best address the needs of medical students.

## Supplementary Information

**Additional file 1: Supplemental Table.** Descriptive statistics including mean, standard deviation, and the minimum and maximum value for each dimension (Satisfaction, Connectedness, School of Medicine Support, Academics, Recourses, and Challenges) for both groups (MS1, MS2). Responses were given the following weights: 4 - Strongly agree, 3 - Agree, 2 - Disagree, 1 - Strongly disagree.

## Data Availability

The datasets generated and/or analyzed during the current study are not publicly available due privacy and confidentiality reasons but are available from the corresponding author on reasonable request.

## Abbreviations

NBME: National Board of Medical Examiners
UCISOM: University of California, Irvine School of Medicine
MS1: First year medical students
MS2: Second year medical students
COVID-19: Coronavirus Disease 2019
USMLE: United States Medical Licensing Examination
